# Considering the *Self* in the Link Between Self-Esteem and Materialistic Values: The Moderating Role of Self-Construal

**DOI:** 10.3389/fpsyg.2019.01375

**Published:** 2019-06-25

**Authors:** Yan Zhang, Skyler T. Hawk

**Affiliations:** ^1^School of Media and Communication, Shenzhen University, Shenzhen, China; ^2^Department of Educational Psychology, The Chinese University of Hong Kong, Hong Kong, China

**Keywords:** self-esteem, self-construal, materialistic values, collectivist culture, experiments

## Abstract

Studies consistently show that materialism might be a strategy people use to cope with low self-esteem. This link might differ among people holding different definitions of the “self” in terms of their relationships with others, however. This research examined the link between self-esteem and materialistic values from the perspective of how people define the self, or their self-construal. In three studies, we explored the moderating role of self-construal in the link between Chinese participants’ self-esteem and materialistic values. Through a self-report survey (Study 1, *N* = 422), experimental manipulation of self-construal (Study 2, *N* = 151), and experimental manipulation of both self-esteem and self-construal (Study 3, *N* = 123), results indicated that self-esteem and self-construal interacted in predicting materialistic values. Specifically, self-esteem negatively predicted materialistic values when interdependent self-construal was low, but not when it was high. We suggest that individuals’ pursuit of materialism under conditions of low self-esteem might depend on how they define the “self.”

## Introduction

Many people consider financial and material success to be their life goals, and place great emphasis on owning high-end products and prestigious brands. Researchers have defined materialistic values as “the importance a person places on possessions and their acquisition as a necessary or desirable form of conduct to reach desired end states, including happiness” (Richins and Dawson, 1992, p. 307). However, too much emphasis on material possessions has consistently shown negative links with lower long-term well-being (e.g., Kasser, 2003, 2005; Dittmar et al., 2014). Therefore, the question of what causes materialism has attracted much research attention. It has been consistently found that self-esteem is a key variable that negatively predicts materialistic values (e.g., Braun and Wicklund, 1989; Hanley and Wilhelm, 1992; Chang and Arkin, 2002; Solberg et al., 2004; Chaplin and John, 2007, 2010). However, prior research has not considered variations in how people define the “self,” which might further lead to different strategies in coping with low self-esteem. We propose that the link between self-esteem and materialistic values might vary based on people’s self-construal, or how they define the “self” in terms of their relationships with others (Markus and Kitayama, 1991).

The negative link between self-esteem and materialistic values has been demonstrated in both survey and experimental studies. Self-report surveys have indicated that self-esteem is inversely correlated with people’s general materialistic values, purchasing expensive brands, or valuing of material possessions (Richins and Dawson, 1992; Mick, 1996; Chaplin and John, 2010; Isaksen and Roper, 2012). Through meta-analysis on 15 correlational studies that included self-evaluation as an indicator of well-being, mainly based on Western samples, Dittmar et al. (2014) indicated that materialistic values are negatively linked to self-appraisals. That is, they concluded that higher levels of materialistic values are associated with lower self-evaluations.

At the same time, despite that materialism is embossed within a broader values system that forms due to many factors and over a relatively long time, and might therefore remain relatively stable (Schwartz, 1992), they might also shift occasionally due to internal states and environmental cues (e.g., Bauer et al., 2012). Experiments focusing on causal effects have also consistently suggested a relationship between self-esteem and materialism, either by directly showing the influence of self-esteem upon materialistic values, or upon consumption tendencies that relate to materialistic values. Manipulation of low self-esteem through experiences of social ostracism promoted materialism (Jiang et al., 2015), and positive social evaluations about personal traits decreased materialistic values (Chaplin and John, 2007). Additionally, Chang and Arkin (2002) manipulated people’s sense of insecurity, a feeling closely linked with low self-esteem, and found elevated levels of materialism among participants. Sivanathan and Pettit (2010) stimulated participants’ need for self-integrity by giving relatively low cognitive ability feedback, and found higher status consumption tendencies. Furthermore, Sheldon and Kasser (2008) tested the impact of three kinds of threats (existential threat, economic threat, and interpersonal threat) on extrinsic goal orientations. After experiencing any of these threats, individuals reported stronger extrinsic goals for money, appearance, and popularity. The studies conveyed consistent information that a state of low self-esteem might increase materialistic values.

Symbolic self-completion theory (Wicklund and Gollwitzer, 1981; Brunstein and Gollwitzer, 1996) provides an explanation for the negative effect of self-esteem on materialistic values. The theory suggests that, under the condition of impaired self-identity, individuals will seek things or engage in activities that can help them regain a positive self-image. Individuals might use the signaling function of material possessions to compensate for threatened self-esteem. The theory aligns with many studies demonstrating the direct negative effect of self-esteem on materialistic values, as the financial success and security represented by material possessions might temporarily boost self-esteem (e.g., Chang and Arkin, 2002).

However, purchasing materialistic possessions as a means to compensate for low self-esteem might vary between individuals, depending on their “self” constructions. Research has implied that people might define the “self” differently in relation to others, and therefore might seek distinct means of fulfilling self-esteem. In other words, peoples’ self-construal might moderate the link between self-esteem and materialistic values.

Individuals with an independent self-construal more strongly emphasize uniqueness and personal achievement, while people with an interdependent self-construal focus more strongly on social connectedness and relations (Markus and Kitayama, 1991). Although some researchers have suggested that everyone possesses both aspects self-construal (e.g., Singelis, 1994), it is also agreed that people typically prioritize one over the other (Markus and Kitayama, 1991), and that being high in one type of construal often implies being low in the other. When individuals focus on pursuing self-expression and personal uniqueness, they are less likely to be relationship-oriented or emphasize connectedness with others (Markus and Kitayama, 1991). Moreover, individuals might also shift to a corresponding independent or interdependent self-construal, respectively, under priming conditions such as searching for words like “I” and “mine” or “we” and “ours” in paragraphs (e.g., Trafimow et al., 1991; Brewer and Gardner, 1996; Gardner et al., 1999; Lin et al., 2008). The contrary effects of the two self-construal primes in these former studies further imply that low interdependent self-construal might suggest independent self-construal (for different opinions, see Hardin et al., 2004; Harb and Smith, 2008). Considering that all the three studies were conducted under Chinese culture, a context where interdependence is emphasized and found to be relatively high (e.g., Li et al., 2006), the paper follows precedents set by other studies implemented in collectivistic cultures (e.g., Fung et al., 2010) by using the terms high and low interdependent self-construal. This terminology is also in line with what the current research wants to communicate, which is that high interdependent self-construal can buffer the relationship between low self-esteem and materialism in collectivistic cultures.

The different nature of the “self” between individuals with high vs. low interdependent self-construal implies different attitudes toward materialistic values when self-esteem is low. Those who emphasize low interdependence enhance and maintain self-esteem through personal success, fulfillment of personal desires, and the validation of their abilities and unique inner attributes. In contrast, the bases of self-esteem for highly interdependent individuals are group harmony and associations with others (Markus and Kitayama, 1991). This distinction might lead individuals low in interdependence be more prone to seek chances for self-expression. Material possessions and prestigious brands might provide a useful and immediate way to convey information about success, status, and personal ability (Eastman et al., 1999; Sivanathan and Pettit, 2010). Therefore, those low in interdependent self-construal might gravitate more toward materialistic values for self-esteem maintenance than individuals high in interdependence.

This proposition is also based on the notion that materialism is indicative of self-centeredness, in line with former research stating that materialistic values were located at the opposite side with collective-orientated values such as family values and religious values in the value system (e.g., Belk, 1985; Schwartz, 1992; Jensen and Jensen, 1993; Kasser and Ryan, 1993; La Barbera and Gürhan, 1997; Rindfleisch et al., 1997; Kasser, 2016). The tension caused by individuals’ materialistic values and the collective-oriented values encouraged by the society contributed an undermined well-being in the long run (Burroughs and Rindfleisch, 2002). The conflict between the two types or values aligns with findings suggesting that collectivism, an overt cultural phenomenon of high interdependent self-construal, is negatively correlated with materialism (Wong, 1997). That is, a stronger emphasis on close relationships and group harmony might be linked to a lower emphasis on materialistic values. Another study reported a negative correlation among Chinese participants between interpersonal harmony and regarding material success as a central goal of life (Sun et al., 2014), further supporting the negative association between collective emphasis and materialism. At the same time, studies have shown that priming concepts of money lead to exchange relationship orientation and emotion suppression in social interactions (Jiang et al., 2013), objectification (i.e., treating others as objects; Teng et al., 2016; Wang and Krumhuber, 2017), and decreased interpersonal harmony (Mead et al., 2013). Such findings imply that materialistic values actually might not be compatible with high quality and close interpersonal relationships. Therefore, those who define the “self” as interdependent and emphasize interpersonal connection are more likely to devalue the importance of material possessions.

The fact that individuals with higher interdependent self-construal emphasize interpersonal connection more, and that materialistic values are more self-oriented, together might contribute to the possibility that individuals differ in employing materialistic values as a coping strategy for low self-esteem. Under self-threat conditions, those who endorse interdependence more would probably be less likely to indicate their uniqueness, while those whose self-definition is less interdependent might express their “self” through material possessions. Indeed, an integral analysis and comparison between lower-interdependent individuals (i.e., Westerners) and higher-interdependent individuals (i.e., Easterners) concluded that highly interdependent individuals experience more social motives (e.g., conformity) but not individually oriented motives (e.g., expressing personal uniqueness) (Bond, 1986; Markus and Kitayama, 1991). A meta-analysis from Dittmar et al. (2014) also indicated that the negative link between materialistic values and self-appraisal, was stronger in societies with higher affective autonomy, a typical indicator in the context of low interdependence. Therefore, individuals who more strongly emphasize interrelationships in defining the “self” (i.e., high interdependent self-construal) might be less likely to become materialistic when experiencing low self-esteem.

According to the reviewed evidence, we expect that the negative link between self-esteem and materialistic values will be stronger for individuals scoring lower in interdependent self-construal than for individuals scoring higher in interdependent self-construal. Our use of participants from mainland China led us to focus primarily on low versus high levels of interdependent self-construal, similar to prior research (e.g., Fung et al., 2010), as this dimension is highly emphasized in the Chinese context. We implemented three studies: In Study 1, we surveyed self-esteem, interdependent self-construal, and materialistic values, using validated self-report scales. In Study 2, we measured self-esteem and manipulated high- vs. low interdependent self-construal to examine the moderating effect of self-construal. In Study 3, we simultaneously manipulated both self-esteem and self-construal in order to examine whether self-construal and self-esteem exert combined causal effects upon materialistic values.

## Study 1

In this study, using a self-report survey, we expected that a negative link between chronic self-esteem and materialistic values would be stronger for individuals scoring lower in interdependent self-construal.

### Methods

#### Participants

Participants were 422 late-adolescents (60.4% female; *M*_age_ = 17.40 ± 0.86) from a senior high school in a relatively less developed, small county in a medium-sized city in the middle of Mainland China. Prior sample size estimation via G^∗^power indicated a minimum of 176 participants (small to medium effect size *f*^2^ = 0.10, power = 0.95), and sensitivity power analysis indicated 95% power to detect an effect size of *f*^2^ = 0.04 (small) with the actual sample size in this survey. Family socioeconomic status (SES; i.e., yearly income) was measured on a 9-point scale, with the following distribution: 1 = less than 3,000 yuan (2.6%), 2 = 3,001–6,000 yuan (6.4%), 3 = 6,001–10,000 yuan (17.3%), 4 = 10,001–30,000 yuan (29.6%), 5 = 30,001–50,000 yuan (17.8%), 6 = 50,001–100,000 yuan (17.3%), 7 = 100,001–150,000 yuan (4.5%), 8 = 150,001–200,000 yuan (3.6%), and 9 = more than 200,001 yuan (0.9%). The mean score of SES was 4.47 (*SD* = 1.59).

#### Measures

##### Self-esteem

The Rosenberg Self-Esteem Scale (RSES: Rosenberg, 1965) measured self-esteem in this study. It was comprised of 10 items answered on a four-point Likert scale (1 = *strongly agree*, 2 = *agree*, 3 = *disagree*, and 4 = *strongly disagree*). An example item is “On the whole, I am satisfied with myself.” We recoded the scores so that higher scores indicated higher self-esteem. Its Cronbach’s alpha was 0.76 in this study.

##### Interdependent self-construal

We used the interdependent subscale of the Self-Construal Scale (SCS) developed by Singelis (1994) to measure interdependent self-construal, which contains 12 items on a single dimension. An example item is: “I often have the feeling that my relationships with others are more important than my own accomplishments.” Participants answered the items on a seven-point Likert scale (1 = *strongly disagree*, 7 = *strongly agree*). The Cronbach’s alpha in this study was 0.71.

##### Materialistic values

Materialistic values in this study were measured by the instrument modified from a six-item scale developed by Opree et al. (2011). The expression of “*children*” in the original items was changed into “*peers*.” On a four-point scale (1 = *no, not at all*, 2 = *no, not really*, 3 = *yes, a little*, and 4 = *yes, very much*), participants responded to items such as “Do you think it is important to own expensive things?” and “Does buying expensive things make you happy?” The Cronbach’s alpha was 0.82 in this study.

##### Demographic variables

Demographic information included age, gender, and socioeconomic status.

#### Procedure

This study was carried out in accordance with the recommendations of Guidelines for Survey and Behavioral Research Ethics, approved by the ethics committee of both the researchers’ home university (Survey and Behavioral Research Ethics of the Chinese University of Hong Kong) and the institutes where they were conducted (the Ethics Committee of Beijing Normal University and the Ethics Committee of Guangxi University), with written informed consent from all subjects (all were older than 16 years old). All subjects gave written informed consent in accordance with the Declaration of Helsinki. Pencil-and-paper instruments were handed out to the participants during after-school self-study time in classrooms, with the instruments organized in the order described above. Oral instructions were given on the aim and the answering method of the study. All participants finished the scales anonymously.

#### Data Analysis

We tested the hypotheses via hierarchical multiple regression. We first standardized the scores of age, SES, self-esteem, interdependent self-construal, and materialistic values, and then calculated the product of self-esteem and interdependent self-construal (SE × SC) for testing the interaction. Gender was coded as 0 = female and 1 = male. In the three-step regression, age, gender, and SES were first entered as control variables, with self-esteem and interdependent self-construal entered in the second step, and the product of self-esteem and interdependent self-construal entered in the third step.

### Results

The first regression step including age, gender, and SES significantly predicted materialistic values, *F*(3, 418) = 2.92, *p* = 0.034, *R* = 0.14, adjusted *R*^2^ = 0.01. This was due to a significant effect of SES (β = 0.10, *t* = 2.06, *p* = 0.040), with higher SES linked to greater materialism. The second step, which added self-esteem and interdependent self-construal, did not significantly improve the model, *F*(5, 416) = 2.29, *p* = 0.045, *R* = 0.16, adjusted *R*^2^ = 0.02; Δ*R*^2^ = 0.02, Δ*F*(2, 416) = 1.34, *p* > 0.250. Neither self-esteem (β = −0.05, *t* = −0.94, *p* > 0.250) or self-construal (β = −0.06, *t* = −1.15, *p* > 0.250) significantly predicted materialistic values. The third step adding the product of self-esteem and self-construal significantly improved the model, *F*(6, 415) = 3.28, *p* = 0.004, *R* = 0.21, adjusted *R*^2^ = 0.03; Δ*R*^2^ = 0.02, Δ*F*(1, 415) = 8.03, *p* = 0.005, *f*^2^ = 0.04, power = 0.94. Although neither self-esteem (β = −0.04, *t* = −0.89, *p* > 0.250) or self-construal (β = −0.04, *t* = −0.76, *p* > 0.250) showed a significant main effect, the interaction [β = 0.14, *t* = 2.83, *p* = 0.005, 95% CI for *B* = (0.03–0.16)] significantly predicted materialistic values (see Table 1).

**Table 1:** Regression model of predictors for material values in Study 1.

	Step 1				Step 2				Step 3			

	*R*^2^ = 0.02; Adjusted *R*^2^ = 0.01; Δ*R*^2^ = 0.02^∗^				*R*^2^ = 0.03; Adjusted *R*^2^ = 0.02; Δ*R*^2^ = 0.01^∗^				*R*^2^ = 0.05; Adjusted *R*^2^ = 0.03; Δ*R*^2^ = 0.02^∗^			
	
Variables	*B*	*SE*	β	95% CI for *B*	*B*	*SE*	β	95% CI for *B*	*B*	*SE*	β	95% CI for *B*
(Constant)	2.13	0.05		[2.03–2.22]	2.13	0.05		[2.04–2.22]	2.11	0.05		[2.02–2.21]
Age	−0.05	0.04	−0.06	[−0.12–0.03]	−0.05	0.04	−0.06	[−0.12–0.03]	−0.05	0.04	−0.07	[−0.13–0.02]
Gender	−0.10	0.08	−0.07	[0.25–0.05]	−0.11	0.08	−0.07	[−0.27–0.04]	−0.11	0.08	−0.07	[−0.27–0.04]
SES	0.08^∗^	0.04	0.10^∗^	[0.004–0.15]	0.08^∗^	0.04	0.10^∗^	[0.01–0.15]	0.07	0.04	0.09	[−0.004–0.14]
SE					−0.04	0.04	−0.05	[−0.11–0.04]	−0.03	0.04	−0.04	[−0.11–0.04]
SC					−0.04	0.04	−0.06	[−0.12–0.03]	−0.03	0.04	−0.04	[−0.10–0.05]
SE × SC									0.10^∗^	0.03	0.14^∗^	[0.03–0.16]

*^∗^p < 0.05.*

**FIGURE 1:**
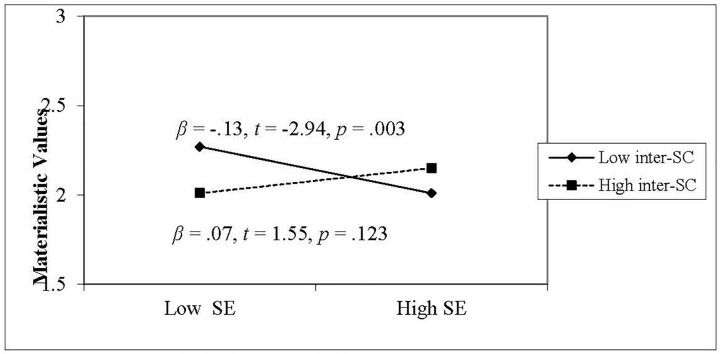
Moderating effect of interdependent self-construal on the relationship between self-esteem and materialistic values in Study 1.

We further used a simple slopes analysis to disentangle the interaction between self-esteem and interdependent self-construal (Aiken and West, 1991). Low and high values in the simple slopes test for self-esteem and interdependent self-construal were set as one standard deviation above and below the mean. As can be seen in Figure 1, there was no relationship between self-esteem and materialistic values when interdependent self-construal was high (β = 0.07, *t* = 1.55, *p* = 0.123). When interdependent self-construal was low, however, the relationship was negative and significant (β = −0.13, *t* = −2.94, *p* = 0.003). The results indicated that, after controlling for age, gender, and SES, self-esteem significantly predicted materialistic values for individuals scoring lower in interdependent self-construal, but not for those scoring higher in interdependent self-construal. These findings were based on chronic measures; the moderating role of self-construal would be further supported if priming a lower versus higher interdependent mindset would again alter the nature of the link between self-esteem and materialism. This was the goal of Study 2.

## Study 2

In Study 2, we manipulated lower vs. higher interdependent self-construal, and examined whether this manipulation moderated a link between participants’ self-reported self-esteem and materialism. We expected that the link between self-esteem and materialistic values would be stronger when lower interdependent self-construal was primed.

### Methods

#### Participants

Participants were 162 college students from mainland China. Five of them skipped too many items (over half), and six provided irregular answers (e.g., marking the maximum or minimum option on all Likert scale items or reporting obvious contradict answers), and were therefore excluded. In the end, 151 participants were included (60.90% female; *M*_age_ = 18.92 ± 1.02), with 77 and 74 belonging to the low and high interdependent self-construal conditions, respectively. Prior sample size estimation via G^∗^power indicated a minimum of 119 participants in total (*f*^2^ = 0.15, power = 0.95), and sensitivity power analysis indicated 95% power to detect an effect size of medium effect size *f*^2^ = 0.12 with the actual sample size in this experiment. The distribution of the participants’ family SES was as follows: 1 = less than 3,000 yuan (6.0%), 2 = 3,001–6,000 yuan (8.6%), 3 = 6,001–10,000 yuan (15.2%), 4 = 10,001–30,000 yuan (17.9%), 5 = 30,001–50,000 yuan (15.9%), 6 = 50,001–100,000 yuan (17.2%), 7 = 100,001–150,001 yuan (11.3%), 8 = 150,001–200,000 yuan (4.0%), and 9 = more than 200,001 yuan (4.0%). The mean score of SES was 4.70 (*SD* = 2.02).

#### Measures

##### Self-esteem

The RSES(Rosenberg, 1965) measured self-esteem in this study, as in Study 1. Its Cronbach’s alpha was 0.83 in this study.

##### Materialistic values

A four-item, unidimensional scale modified from Kasser (2005) measured materialistic values on a seven-point scale (1 = *strongly disagree*, 7 = *strongly agree*). This scale was developed from several existing materialism scales from Richins and Dawson (1992), Cohen and Cohen (1996), Kasser and Ryan (1996), and Schor (2003, Unpublished), and was believed to reflect the most important components in existing instruments (Kasser, 2005). The unsuitable phrase of “*when I grow up*” for college students in items like “*When I grow up, I want to own lots of money*” was removed. The Cronbach’s alpha was 0.62 in the current study.

##### Demographic variables

Demographic information included age, gender, and SES. SES was measured by family income of the past year by the same scale as in Study 1.

#### Procedure

Participants completed pencil and paper questionnaires in class. They first reported their self-esteem, and moved on to the high- or low interdependent self-construal priming task.

The priming task was reading essays and answering questions used in prior studies (Gardner et al., 1999; Sui and Han, 2007). In each condition, participants read two stories describing a trip to the countryside, with each of the two-paragraph essays containing high interdependent pronouns (e.g., *we, us, and ours*) or low interdependent pronouns (e.g., *I, me, and mine*). The essays described what “I”/“we” did, what “I”/“we” saw, and other stories happened during the trip. All the contents of the essays were the same between the two conditions except the pronouns. Participants were required to carefully read the essay, count the total number of pronouns, and circle them in the essay.

Participants then completed the materialistic values scale and demographic information. All participants received the high or low interdependent self-construal priming material randomly, and data comparison showed no difference between the two groups on age, gender distribution, SES, or self-esteem level.

#### Data Analysis

We tested the hypotheses via a hierarchical multiple regression procedure. We first standardized the scores of age, SES, and self-esteem. We dummy coded self-construal as −1 (low interdependent self-construal) and +1 (high interdependent self-construal) and then calculated the product of self-esteem and interdependent self-construal. Gender was coded as 0 = female and 1 = male. In the three-step regression, age, gender, and SES were first entered as control variables, with self-esteem and interdependent self-construal entered in the second step, and the product of self-esteem and self-construal entered in the third step.

### Results

The first regression step including age, gender, and SES was not significant, *F*(3, 147) = 0.37, *p* > 0.250, *R* = 0.10, *R*^2^ = 0.01. In the second step, the addition of self-esteem and interdependent self-construal significantly improved the model (Δ*R*^2^ = 0.05, Δ*F*(2, 145) = 3.88, *p* = 0.023), although the model was still not significant, *F*(5, 145) = 1.79, *p* = 0.120. Self-esteem significantly predicted materialistic values (β = −0.22, *t* = −2.69, *p* = 0.008), but self-construal did not (β = −0.06, *t* = −0.77, *p* > 0.250). In the third step, the interaction of self-esteem and self-construal (SE × SC) was added to the model. Results indicated that the model became significant, and also showed a trend toward significant improvement over the second step [*F*(6, 144) = 2.12, *p* = 0.054, *R* = 0.29, adjusted *R*^2^ = 0.04, Δ*R*^2^ = 0.02, *p* = 0.058, *f*^2^ = 0.08, power = 0.83]. The interaction of self-esteem and interdependent self-construal showed a trend toward predicting materialistic values, β = 0.15, *t* = 1.91, *p* = 0.058 (see Table 2).

**Table 2:** Regression model of predictors for material values in Study 2.

	Step 1				Step 2				Step 3			
	
	*R*^2^ = 0.01; Adjusted *R*^2^ = −0.01; *R*^2^ = 0.01				*R*^2^ = 0.06; Adjusted *R*^2^ = 0.03; *R*^2^ = 0.05^∗^				*R*^2^ = 0.08^∗^; Adjusted *R*^2^ = 0.04^∗^; Δ*R*^2^ = 0.02^†^			
	
Variables	*B*	*SE*	β	95% CI for *B*	*B*	*SE*	β	95% CI for *B*	*B*	*SE*	β	95% CI for *B*
(Constant)	4.67	0.11		[4.45–4.89]	4.65	0.11		[4.43–4.87]	4.65	0.11		[4.43–4.86]
Age	−0.08	0.09	−0.07	[−0.26–0.11]	−0.09	0.09	−0.09	[−0.27–0.09]	−0.08	0.09	−0.08	[−0.26–0.09]
Gender	0.11	0.18	0.05	[−0.25–0.47]	0.14	0.18	0.07	[−0.21–0.50]	−0.16	0.18	−0.08	[−0.19–0.51]
SES	−0.06	0.09	−0.06	[−0.24–0.11]	−0.02	0.09	−0.02	[−0.20–0.15]	−0.02	0.09	−0.02	[−0.19–0.15]
SE					−0.23^∗^	0.09	−0.22^∗^	[−0.40− (−0.06)]	−0.23^∗^	0.09	−0.22^∗^	[−0.39− (−0.06)]
SC					−0.07	0.08	−0.06	[−0.23–0.10]	−0.07	0.09	−0.06	[−0.23–0.10]
SE x SC									0.16^†^	0.09	0.15^†^	[−0.006–3.24]

*^∗^p < 0.05, ^†^p < 0.06.*

**FIGURE 2:**
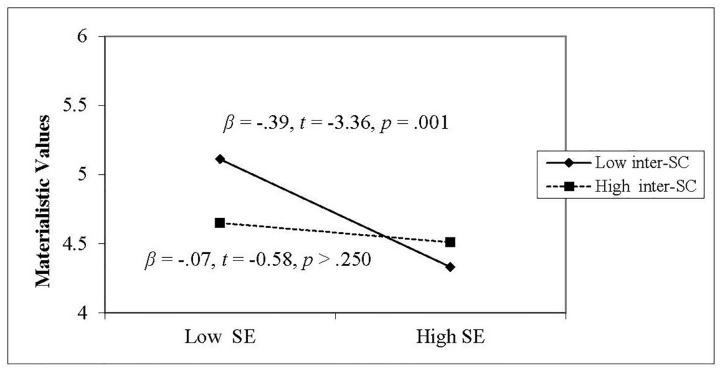
Moderating effect of interdependent self-construal on the relationship between self-esteem and materialistic values in Study 2.

We further used a simple slopes analysis (Aiken and West, 1991) to clarify the nature of the interaction. Low and high values in the simple slopes test were set as one standard deviation above and below the mean. As seen in Figure 2, when high interdependent self-construal was primed, there was no relationship between self-esteem and materialistic values (β = −0.07, *t* = −0.58, *p* > 0.250). When low interdependent self-construal was primed, however, the relationship was negative and significant (β = −0.39, *t* = −3.36, *p* = 0.001). Thus, the negative relationship between self-esteem and materialistic values under conditions of low interdependent self-construal was replicated. We then conducted a final experiment to examine the combined causal effects from both self-esteem and interdependent self-construal upon materialistic values.

## Study 3

In Study 3, we employed a bogus “Image Personality Test” to manipulate self-esteem and self-construal simultaneously, and test their combined effects on materialistic values. We again predicted that a combination of low self-esteem and low interdependent self-construal would yield the highest scores of materialistic values.

### Methods

#### Participants

Participants were 123 college students (73.8% female, one missing; *M*_age_ = 21.40 ± 2.52, three missing) from mainland China. Prior sample size estimation via G^∗^power indicated a minimum of 119 participants in total (medium effect size *f* = 0.26, power = 0.80), and sensitivity power analysis indicated 80% power to detect an effect size of *f* = 0.25 with the actual sample size in this experiment. They were randomly assigned to the high self-esteem/high interdependent self-construal group (HSE-HSC; *n* = 28), the high self-esteem/low interdependent self-construal group (HSE-LSC; *n* = 31), the low self-esteem/high interdependent self-construal group (LSE-HSC; *n* = 36), and the low self-esteem/low interdependent self-construal group (LSE-LSC; *n* = 28). Their family SES (one missing) was distributed as follows: 1 = less than 5,000 yuan (0%), 2 = 5,001–10,000 yuan (4.9%), 3 = 10,001–30,000 yuan (10.6%), 4 = 30,001–50,000 yuan (17.1%), 5 = 50,001–100,000 yuan (25.2%), 6 = 100,001–150,000 yuan (22.0%), 7 = 150,001–200,000 yuan (8.1%), 8 = 200,001–300,000 yuan (7.3%), 9 = 300,001–500,000 yuan (2.4%), and 10 = more than 500,001 yuan (1.6%). The mean score of SES was 5.25 (*SD* = 1.74).

#### Measures

##### Demographic variables

Demographic information included age, gender, SES (family income of the past year, see the participants section).

##### Manipulation check

**FIGURE 3:**
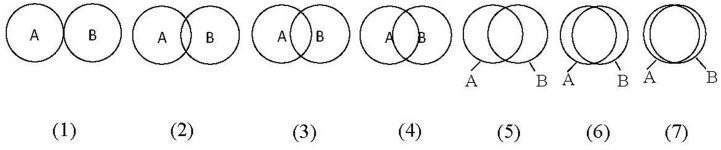
Figures for self-esteem manipulation check in pilot study of Study 3, and for self-construal manipulation check in formal Study 3.

We included a single-item scale modified from Aron et al. (1992) to assess the manipulation of self-construal. Participants were required to circle the picture which best described their relationship with others. In our study, we used social groups as the object for relationship consideration, with higher scores indicating higher interdependence. The instruction was: *If circle A represents your members of your social groups and circle B represents your “self,” how connected do you feel to members of your social groups at the moment?* (see Figure 3).

A similar scale was employed for self-esteem manipulation check in a pilot study before the formal experiment (but was not used in the formal experiment). We asked participants to choose the picture that best described the distance between their current and ideal “self” with the instruction: *If circle A represents your “current self” and circle B represents your “ideal self,” how would you describe their relationship at the moment?* In our study, higher self-esteem scores were given (from 1 to 7) when participants selected a picture with a greater overlap between their current and ideal selves (Figure 3). We separated the self-esteem manipulation check in a pilot study to avoid confusion in understanding with the self-construal manipulation check in formal experiment.

##### Materialistic values

The same four-item unidimensional scale as in Study 2, modified from Kasser (2005) measured materialistic values on a seven-point scale (1 = *strongly disagree*, 7 = *strongly agree*). The Cronbach’s alpha was 0.65 in the current study.

#### Procedure

Participants finished the task in lab in both the pilot study and the formal study in Study 3. We created a bogus “Image-Personality Test” in order to manipulate the independent variables in this study. Before the formal experiment, we conducted a pilot study to check the efficacy of the program in manipulating self-esteem.

In the pilot study, we recruited a total of 177 participants (*M*_age_ = 19.35 ± 1.16, 63.8% female), who were randomly designated into high (*n* = 91) or low (*n* = 86) self-esteem conditions, with no difference on age, gender, and SES between the groups. They first completed the bogus “Image-Personality Test.” in which they were required to choose one out of four pictures composed of different colors, lines, and patterns, but also shared some common characteristics in every trial and therefore could be viewed as ostensibly being linked to certain personality traits. After a total of 24 trials, participants received specific feedback for each of five personality traits (including conscientiousness, agreeableness, openness, self-control, and flexibility), with a bogus mean score of all participants also presented for reference.

**FIGURE 4:**
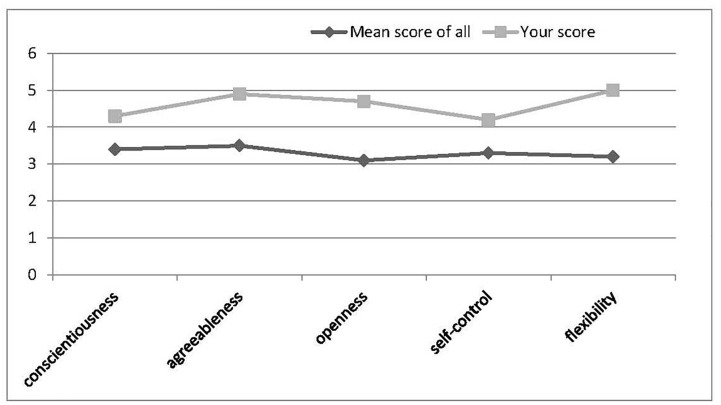
Feedback for bogus “Image Personality Test” in the high self-esteem condition (Study 3).

Self-esteem was manipulated by giving feedback showing either high scores (high SE) or low scores (low SE) relative to the “average” score of other participants. The feedback for the high self-esteem condition was as follows, with a figure presented at the same time (Figure 4):

“In this test, each of the five traits was reflected on 6 items, and the score varied from 0 to 6. According to the test on your traits just now, you generally received high scores (with the mean score higher than 78% participants in our experiment, on average) on the five aspects we measured. Below is your score on each aspect.”

**FIGURE 5:**
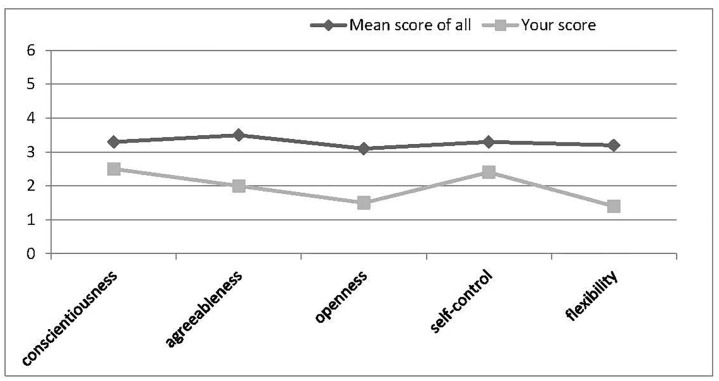
Feedback for bogus “Image Personality Test” in the low self-esteem condition (Study 3).

The feedback for low self-esteem condition was (Figure 5):

“*In this test, each of the five traits was reflected on 6 items, and the score varied from 0 to 6. According to the test on your traits just now, you generally received low scores (with the mean score lower than 78% participants, or only higher than 22% others in our experiment, on average) on the five aspects we measured. Below is your score on each aspect*.”

After the participants read the feedback, they answered the question for the manipulation check on self-esteem as described in measures section. After controlling age, gender, and SES, a *t* test in SPSS indicated that the high self-esteem group (*M* = 3.78 ± 1.43) and low self-esteem group (*M* = 3.29 ± 1.42) reported significantly different distances between their current and ideal selves, *t*(175) = 2.30, *p* = 0.023, 95% CI for the mean differences was [0.07 – 0.92] without zero included, suggesting that the “Image-Personality Test” was effective in manipulating self-esteem and therefore could be used in formal experiment.

In the formal study, we further added the manipulation of self-construal in the test. An initial cover story was given explaining that the study was composed of tasks from several unrelated projects, and the whole study contained two parts: the first part was a newly developed “image-personality test” (the manipulations of self-esteem and self-construal), and the second part was a survey on college students’ lifestyles. After providing demographic information, participants were designated randomly into one of the four manipulation groups: HSE-HSC, HSE-LSC, LSE-HSC, and LSE-LSC. Therefore, this was a 2 (self-esteem: high vs. low) × 2 (interdependent self-construal: high vs. low) factorial design. No significant differences on gender distribution, age, or SES were found between the four groups. The participants first completed the bogus “Image-Personality Test” as they did in our pilot study. In the feedback stage of the test, participants received the specific feedback on their scores on each of the five traits used in the pilot study, and additionally received feedback on the extent to which they might share the personality characteristics with other members of their social groups.

Interconnectedness to others (Aron et al., 1992) is important for individuals with high interdependent self-construal and similarity produces perceptions of closeness (Liviatan et al., 2008; Boot and Pecher, 2010). Evidence from research on interpersonal behavior has supported the associations between similarity and interdependent self-construal. For example, non-conscious behavioral mimicry, a signal of interpersonal similarity, actually results in participants’ shifts toward more interdependent self-construal (Van Baaren et al., 2003; Ashton-James et al., 2007). Therefore, a high similarity feedback could arouse perceptions of interconnectedness and prime an interdependent mindset.

**FIGURE 6:**
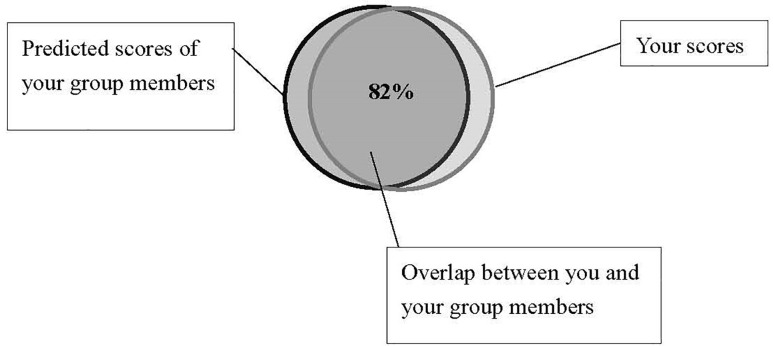
Feedback for bogus “Image Personality Test” in the high interdependent self-construal condition (Study 3).

The feedback paragraph for high interdependent self-construal group was as follows (see also Figure 6):

“*The pattern of your answers suggests a very high likelihood (up to 82%) that many members of the social groups that you are in would receive scores that are similar to your own, namely that you and they share the same tendencies on these personality traits. Two circles below reflect the patterns, and the overlapping part indicates the shared personality characteristics between you and your group members*.”

**FIGURE 7:**
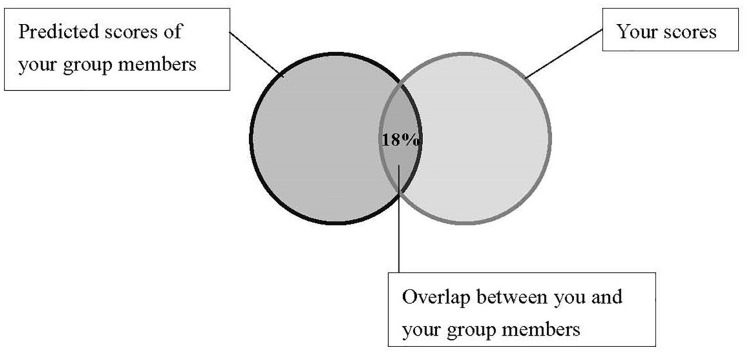
Feedback for bogus “Image Personality Test” in the low interdependent self-construal condition (Study 3).

The feedback for low interdependent group was as follows (see also Figure 7):

“*The pattern of your answers suggests a very low likelihood (18% or less) that members of the social groups that you are in would receive scores that are similar to your own, namely that you are quite different from them on these personality traits. Two circles below reflect the patterns, and the small overlap indicates that you only shared very limited personality characteristics with your group members*.”

After the participants received the feedback, they answered questions for the manipulation check of self-construal, and then the self-report items for materialistic values. After the task, they were asked for their opinions about the aim of the study, thanked with a reward, and debriefed.

#### Data Analysis

Data analysis was conducted in SPSS. We first dummy coded the conditions of high- and low self-esteem as +1 and −1, and the conditions of high- and low- interdependent self-construal as +1 and −1. Gender was coded as 0 = female and 1 = male. We then checked that there was no difference on gender distribution, SES, and age between the four groups. We used a two-way ANOVA to check the main effects of self-esteem and self-construal, and their interaction on materialistic values.

### Results

#### Manipulation Check of Self-Construal

Manipulation check on self-construal was conducted with a *t* test. It indicated that the high interdependent self-construal group (*M* = 4.45 ± 1.46) and low interdependent self-construal group (*M* = 3.69 ± 1.45) reported significantly different self-construal, *t*(121) = 2.89, *p* = 0.005, 95% CI for mean difference = (0.24–1.28), suggesting that the added feedback manipulation of self-construal was effective.

#### Interaction Effect Between Self-Esteem and Self-Construal

We used a two-way ANOVA to test the main effects and the interaction of self-esteem and self-construal manipulations on materialistic values, with age, gender, and SES entered as covariates. Neither the self-esteem [*F*(1,113) = 2.16, *p* = 0.145, $\eta_{p}^{2}$ = 0.02], or the interdependent self-construal [*F*(1,113) = 1.11, *p* = 0.295, $\eta_{p}^{2}$ = 0.01] manipulations showed direct effects on materialistic values. However, the interaction between self-esteem and self-construal showed a significant effect on materialistic values [*F*(1,113) = 4.48, *p* = 0.036, $\eta_{p}^{2}$ = 0.04, power = 0.64].

**FIGURE 8:**
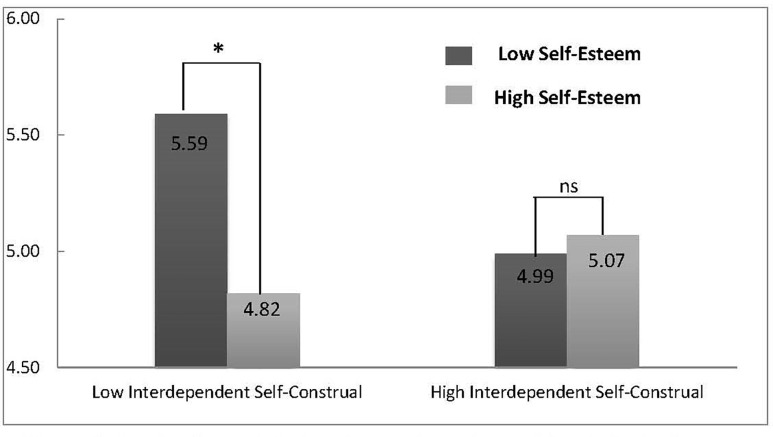
Levels of materialistic values under each condition in Study 3. ^∗^*p* < 0.05.

A simple effects test was used to examine specific differences in materialistic values between the four experimental conditions. It indicated that low self-esteem priming (*M*_lse–hsc_ = 4.99 ± 0.97) and high self-esteem priming (*M*_hse–hsc_ = 5.07 ± 1.24) did not lead to significantly different materialistic values within the high interdependent self-construal condition, *F* (1, 113) = 0.21, *p* = 0.647, $\eta_{p}^{2}$ = 0.002. However, higher materialistic values were observed for the low self-esteem condition (*M*_lse–lsc_ = 5.59 ± 0.77) than for the high self-esteem condition (*M*_hse–lsc_ = 4.82 ± 1.01) when low interdependent self-construal was primed, *F* (1, 113) = 6.22, *p* = 0.014, $\eta_{p}^{2}$ = 0.05, power = 0.72. Levels of materialistic values of the four groups are presented in Figure 8.

Our simultaneous manipulation of both self-esteem and self-construal further demonstrated their combined causal effects upon materialistic values. We found that priming low self-esteem increased materialistic values for the individuals who were primed with low interdependent self-construal, but there was no effect of self-esteem on materialistic values for the participants primed with high interdependent self-construal.

## General Discussion

In three studies, we found that people’s higher or lower interdependent self-construal, both in terms of chronic states and temporary priming conditions, moderated their emphasis on materialistic values when experiencing self-threat. This research challenges the conventional view that low self-esteem is a stable mechanism for high materialistic values, by showing that the link only holds for individuals with lower levels of interdependent self-construal.

Symbolic self-completion theory (Wicklund and Gollwitzer, 1981) can explain people’s materialistic behaviors under conditions of low self-regard. Our research, however, demonstrated that the effect of self-esteem on materialistic values might further depend on how people define the self. Materialism might be a way to fulfill individuals’ goals for uniqueness and validation of personal attributes. Therefore, when experiencing low self-esteem, their valuation of material possessions would be elevated. For the individuals who define the “self” more in terms of relationships with their social contexts and close others, however, intense self-expression through material possessions might not be the most efficient way to maintain self-esteem. This distinction implies that, although all people are motivated to maintain self-esteem (Leary and Baumeister, 2000), they might enact different strategies depending on where their sense of “self” comes from. Prestigious brands might not be sufficient to buffer low self-esteem for those who strongly emphasize their interconnections with others. This appears to be the case regardless of whether self-esteem and self-construal are chronically measured or experimentally manipulated.

Despite that the current research demonstrated a buffering effect of high interdependent self-construal to low self-esteem individuals’ materialism, the relatively high level of materialistic values in the collectivistic Chinese society calls for more explanation. While a higher interdependent self-construal would protect individuals from materialism under conditions of low self-esteem, the generally high materialistic values of a society could be a result of many factors. As a rapidly developing country, China has experienced dramatically increasing economic power in recent years. On a national level, this could lead to value transformations toward materialism (Inglehart et al., 1998; cf. Zhou, 2009). Other factors such as increased marketing efforts across the country and emphasis on hierarchical status in the culture might also contribute to higher materialistic norms and values (Sangkhawasi and Johri, 2007; Goldsmith and Clark, 2012).

The present research offers several novel contributions. Theoretically, we showed that low self-esteem does not hold an absolute relationship with high materialistic values, as implied by prior studies (e.g., Richins and Dawson, 1992; Chang and Arkin, 2002; Chaplin and John, 2007). Methodologically, we used a new method of feedback to simultaneously manipulate both self-esteem and self-construal in Study 3. The method we created showed advantages of (1) being able to combine or embed the manipulations of multiple variables, and (2) directly present participants’ attributes in terms of sharing similarities with others, and therefore could be useful for future studies. This research also holds practical implications, especially for interventions aimed at reducing materialistic values. Specifically, as individual holding lower interdependent self-construal are prone to use materialism to cope with threats to the “self” and regain positive self-perception, other methods of providing space for self-expression and demonstrating uniqueness to promote self-esteem might be effective, and would hopefully reduce their materialism. At the same time, emphasis on interconnectedness with others when facing self-threats might also buffer the tendency to adopt materialism. Considering that materialism could potentially impact people’s long-term life satisfaction (Kasser, 2005), educators, practitioners, and parents might consider characteristics of the “self” when planning and implementing interventions aimed at decreasing materialistic values.

Our studies were conducted in three different cities in mainland China that differ in economic levels and local micro-cultures. Participants also spanned from high school students to university students, and varied in their family SES. We also altered our study methods, from self-report questionnaires to a 2 × 2, fully experimental design. These variations in samples and methods, but not in our primary finding, speak to the robustness of these results.

Nevertheless, there are limitations that can be improved in future research. Firstly, the results were based on Chinese participants, who are embedded in a relatively high interdependent cultural context and might hold relatively higher interdependent self-construal, as a whole. Although we manipulated self-construal in Studies 2 and 3, it is possible that people’s default mode of self-construal might still play a role in the process. Therefore, more research is needed to replicate the findings cross-culturally. Secondly, the sample sizes in Study 2 and 3 were relatively small, and were not balanced between groups after excluding those reporting suspicions about the study and manipulations. Including more participants and working to ensure a more balanced distribution between experimental conditions would contribute to further confidence in those results. Thirdly, the mechanisms between self-esteem and materialistic values need more exploration. Individuals showing lower interdependence emphasize autonomy and personal goals, and are more likely to show self-protective and self-enhancement tendencies following personal failures (Brockner and Chen, 1996). Therefore, an underlying self-enhancement process might be further examined. Highly interdependent individuals’ extra emphasis on social connection, including interpersonal motivations such as belongingness needs or collective self-esteem, might factor more heavily into their materialism. Interpersonal influences, such as conformity in consumer decision-making, might therefore be a greater source of materialism for individuals in collectivist cultures (Hui and Triandis, 1986; Choi and Geistfeld, 2004). In conclusion, our research indicated that the link between materialistic values and self-esteem might be dependent on how individuals define the “self.”

## Author Contributions

YZ contributed substantially to the study concept development, design of the study, analysis and interpretation of the data of the study, and also drafted the work for important intellectual content. SH made substantial contributions to design of the study, the analysis and interpretation the data of the study, and also drafted and revised the work for important intellectual content.

## Conflict of Interest Statement

The authors declare that the research was conducted in the absence of any commercial or financial relationships that could be construed as a potential conflict of interest.
